# Topological motifs populate complex networks through grouped attachment

**DOI:** 10.1038/s41598-018-30845-4

**Published:** 2018-08-23

**Authors:** Jaejoon Choi, Doheon Lee

**Affiliations:** 1 Bio-Synergy Research Center, 291 Daehak-ro, Yuseong-gu, Daejeon, Republic of Korea; 2 Department of Genetics, Harvard Medical School, 77 Avenue Louis Pasteur, Boston, Massachusetts United States of America; 3 Department of Bio and Brain Engineering, Korea Advanced Institute of Science and Technology (KAIST), 291 Daehak-ro, Yuseong-gu, Daejeon, Republic of Korea

## Abstract

Network motifs are topological subgraph patterns that recur with statistical significance in a network. Network motifs have been widely utilized to represent important topological features for analyzing the functional properties of complex networks. While recent studies have shown the importance of network motifs, existing network models are not capable of reproducing real-world topological properties of network motifs, such as the frequency of network motifs and relative graphlet frequency distances. Here, we propose a new network measure and a new network model to reconstruct real-world network topologies, by incorporating our Grouped Attachment algorithm to generate networks in which closely related nodes have similar edge connections. We applied the proposed model to real-world complex networks, and the resulting constructed networks more closely reflected real-world network motif properties than did the existing models that we tested: the Erdös–Rényi, small-world, scale-free, popularity-similarity-optimization, and nonuniform popularity-similarity-optimization models. Furthermore, we adapted the preferential attachment algorithm to our model to gain scale-free properties while preserving motif properties. Our findings show that grouped attachment is one possible mechanism to reproduce network motif recurrence in real-world complex networks.

## Introduction

Researchers have developed network models for real-world systems such as protein-protein interactions (PPIs), author collaborations, the World Wide Web (WWW), and social networks in order to analyze the relationship between the functions and structures in those real-world systems. Each real-world system has its own properties that can be described in terms of network measures such as network centralities, average path length, and degree distribution. The three classic models for describing real-world properties are the Erdös–Rényi (ER)^1^, small-world (SW)^2^, and scale-free (SF)^3^ models. Although several variations of these standard network models and other models have been proposed, these three models are still widely used in network analysis^4–6^. Recently, two hyperbolic geometrical models have been developed: popularity-similarity-optimization (PSO)^7^, and nonuniform popularity-similarity-optimization (nPSO)^8,9^ models. These models have been proved to be able to reproduce real-world properties such as clustering, small-worldness, power-lawness, rich-clubness and community structure^8,10,11^.

Network motifs are recurrent and statistically significant partial subgraphs or patterns^12^, and graphlets are small connected non-isomorphic induced subgraphs^13^. Although these two concepts are defined slightly differently, they are commonly used interchangeably. Various studies on topological measures of networks have highlighted the importance of network motifs and graphlets in analyzing real-world networks properties, including scale-free, geometric, complex, or high-order networks^13–19^. Some proposed topological measures of network motifs and graphlets include frequency of network motif^14^, graphlet degree distributions (GDD)^15^, and relative graphlet frequency distances (RGF-distances)^13^.

Here we suggest a new network measure which represents real-world topological properties and a new network model incorporating the grouped attachment (GA) to resemble real-world topological properties of network motifs. To validate the GA models, we show that the GA model networks have motif properties more similar to real-world networks than other tested conventional models.

## Results

### Network motifs in network models

While recent studies have shown the importance of analyzing network motifs and graphlets^20–25^, current network models are not capable of reproducing real-world topological properties of network motifs. To show the incapability of the network models, we examined network motif frequencies of the canonical Wnt signaling pathway (see Supplementary Fig. S1) in the NCI (National cancer institute)/Nature database. For the given real network, we generated corresponding model networks by the network models (ER, SW and SF). For each network model, 100 random networks are generated with input parameters optimized from the real network (See method session for detailed description of network generation), and directed 3-node motif frequency distributions of the networks are examined.

**Figure 1:**
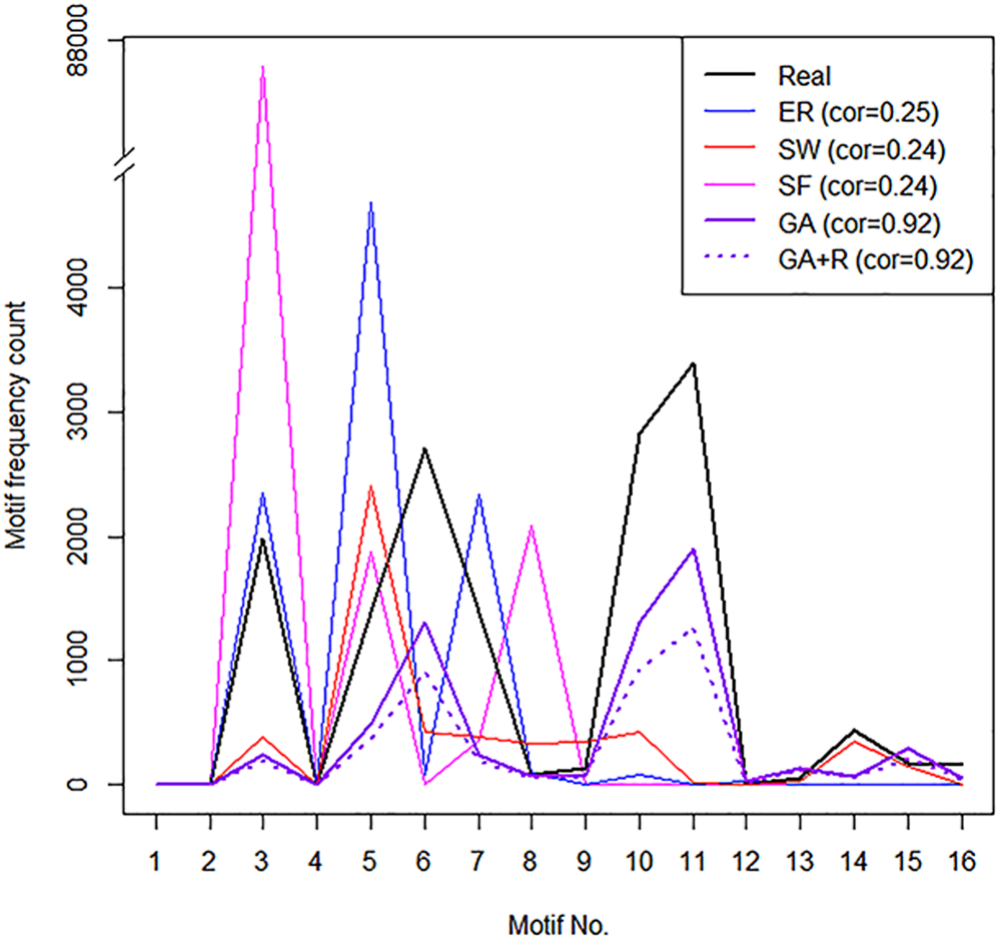
Motif property analysis result of canonical Wnt signaling pathway in the NCI/Nature database. Directed 3-node motif frequency of a real-world network and its corresponding model networks of existing network models (ER, SW and SF) and our models (GA and GA + R). The horizontal axis is directed 3-node motif number, and the vertical axis is motif frequency count. Pearson correlation coefficients between the real network and corresponding model networks are stated in legends. GA and GA + R models show higher similarity of motif frequencies to the real-world network compared to other network models. Legend: ER = Erdös–Rényi; SW = Small-world; SF = Scale-free; GA = Grouped attachment.

As we show in Fig. 1, none of previous network models (ER, SW and SF) reproduced a motif frequency distribution of the real-world network (black line) compared to our proposed model (GA and GA + R) networks (purple lines). GA model networks have significantly high correlations (correlation coefficients = 0.92) with the real network compared to previous network models. This result shows that previous network models have quite different network motifs from the real network, and our proposed model networks have higher network motif similarity to the real network compared to other network models.

### Co-neighborness of a graph

**Figure 2:**
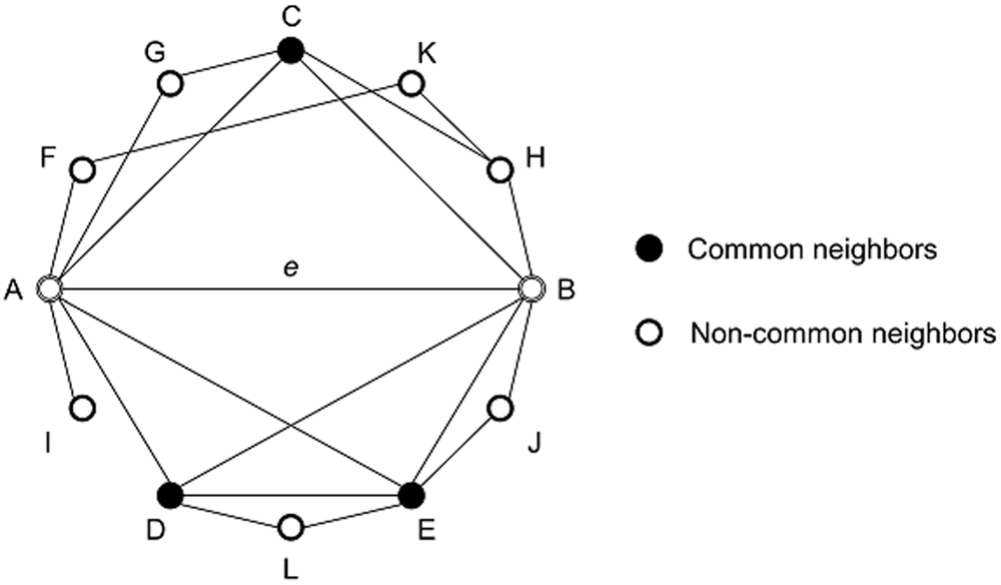
Common neighbors and Jaccard’s index of an edge. The figure shows an example of common neighbors. Among all neighbor nodes (C, D, E, F, G, H, I, J, K and L) of edge *e* (A-B), nodes which are adjacent to both of A and B are co-neighbor nodes (C, D and E). Jaccard’s index of edge *e* (A-B) is 3/10 = 0.3.

To begin to address a new network model which can reproduce real-world topological properties of network motifs, we focus on the concept of common neighbors index^26^ (Fig. 2). Common neighbors index represents the likelihood that two nodes interact increases if overlap of their first-node-neighbors (adjacent nodes) increases. Several studies^27–32^ claimed that nodes in the same community or cluster have high similarities and common neighbors index are highly related to community or cluster structures in networks. Nodes in the same cluster can be clustered based on various criteria such as vertex connectivity or neighborhood similarity^28^, and nodes in a community structure show high similarities^29^. We assumed that neighborhood similarities of related nodes could be a key solution to reproduce real-world topological properties of network motifs. Therefore, we suggest a new network measure, co-neighborness, which shows neighborhood similarity of nodes in the network.

Let *G* = (*V*, *E*) be a graph with node set *V* and edge set *E*. We defined co-neighborness of a graph as an average value of Jaccard’s indices^33,34^ of edges in the graph:

1 $$cn(G)=\frac{1}{|E|}{\sum }_{e(u,v)}^{E}JC(u,v),$$

where two nodes *u*, *v* ∈ *V* are connected by an edge *e* *∈* *E*, and *JC(u, v)* is a Jaccard’s index of node *u* and *v*. Jaccard’s index is normalized common neighbors index^33,34^. (See method section for the equation of Jaccard’s index).

**Figure 3:**
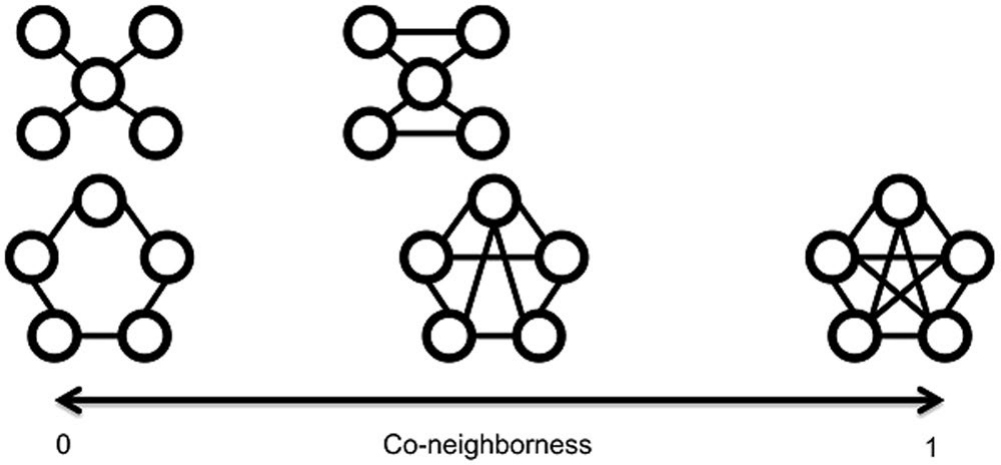
Co-neighborness of a graph. Example graphs are illustrated with their co-neighborness. A graph with no triangle subgraph (fully connected 3-node subgraph) has co-neighborness as 0. A fully connected graph has co-neighborness as 1.

The co-neighborness of a graph has range from 0 to 1. If the co-neighborness is close to 0, few common neighbors of an edge (two adjacent nodes) exist. If the co-neighborness is close to 1, most adjacent nodes of an edge (two adjacent nodes) are common neighbors. A graph with no ‘triangle subgraph (fully connected 3-node subgraph)’ has co-neighborness as 0. A fully connected graph has co-neighborness as 1 (Fig. 3).

There are several existing network measures (see Supplementary Note) which are related to co-neighborness such as graph density or average/global clustering coefficient^35^. These measures can have high correlation with co-neighborness, but have different values as co-neighborness has a distinctive definition (see Supplementary Table S3).

### Grouped attachment

**Figure 4:**
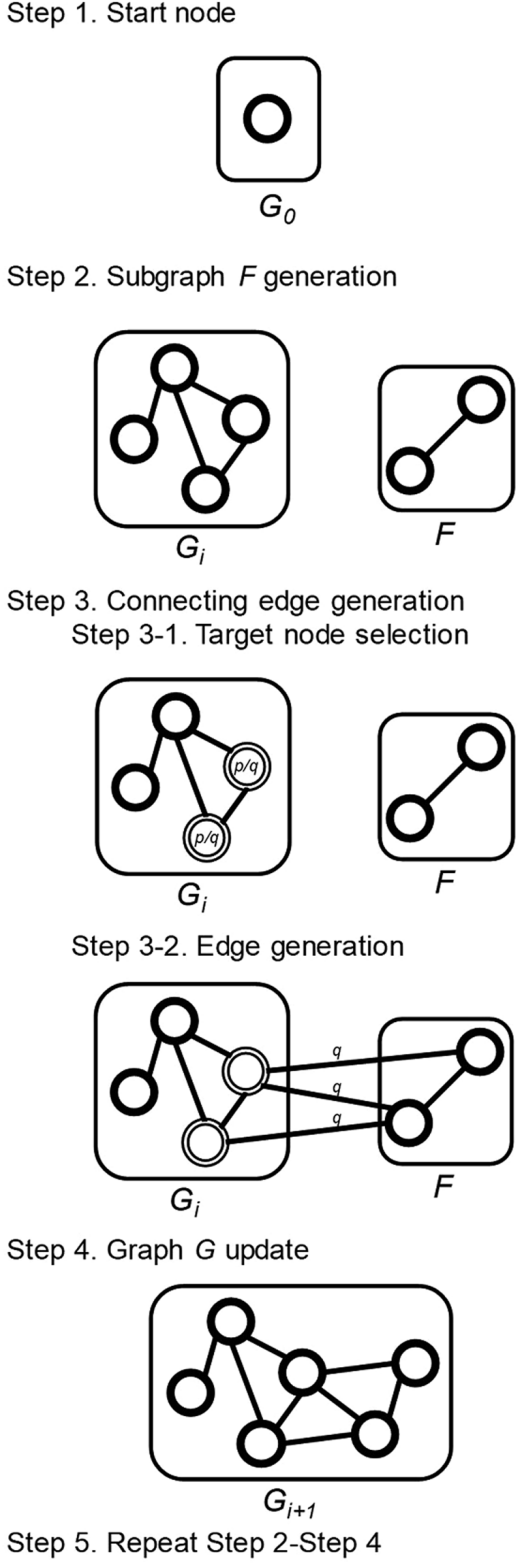
Network generation procedures of grouped attachment model. The GA model gets three input parameters; *n*: node count, *p*: edge probability, and *q*: groupness probability. Like the preferential attachment model, attachments are processed repeatedly.

To reflect the real-world co-neighborness property, we consider the following grouped attachment procedure (Fig. 4). We get three values as input parameters: (1) *n* as a node count, (2) *p* as an edge probability (0 < *p* < 1) and (3) *q* as a groupness probability (*p* < *q* < 1, see Supplementary Note). Starting from a single node graph *G*_0_, at every repeat we create and add a highly interconnected graph *F*, which is generated by an edge extension model (see Supplementary Note) with an input probability *q*. Then, we create edges that connect the nodes in the graph *F* to the nodes in the graph *G*. Among the nodes in the graph *G*, we select nodes with a probability *p/q* to be connected, and for every selected nodes, we connect to the nodes in the graph *F* with a probability *q* (*p* < *q* < 1). We repeat the procedures until the graph *G* has *n* nodes.

By following the procedures above, we can generate edges connecting the graph *G* to the graph *F* with a probability *p* in total. Compared to a random selection of edges with a probability *p*, the procedures guarantee high neighborhood similarities of nodes in the graph *F*, when *q* has a high value. If *q* has a low value, edges are created almost similar as random selection with probability *p*, which leads to generate a graph similar to the ER model.

The grouped attachment has similarities and differences with the preferential attachment (SF model)^3^. Indicated by their names, both models have growing characteristics which is implemented through attachments. Both models start with a small number of nodes (one node for grouped attachment), and at every repeat a new group of nodes (one node for preferential attachment) are added with edges that link the new nodes to the nodes already present in the system. However, grouped attachment does not preferentially attach nodes, which means nodes are not connected depending on degree of nodes. Instead, grouped attachment adds group of nodes at each repeat, while preferential attachment adds a single node at each repeat.

**Figure 5:**
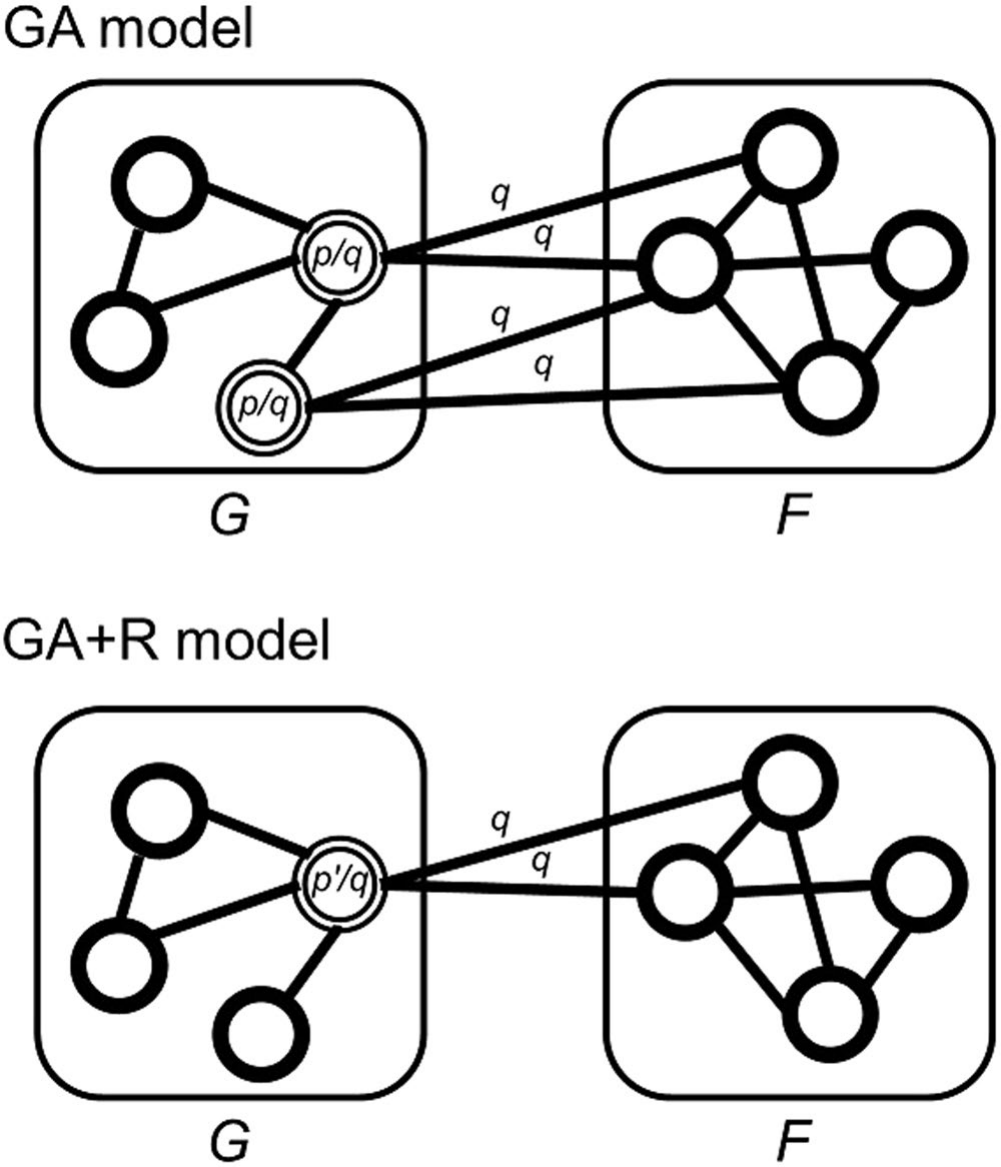
Edge generation procedures of GA and GA + R model. The figure shows an example of edge generation procedures (Step 3 of Fig. 4) of both models. Both GA and GA + R models are supposed to have edge density as *p*, while edge density of graph *F* is not guaranteed to be *p*. To adjust total edge density to be *p*, the edge generation procedure of GA + R model selects target nodes with probability of *p*′*/q*, instead of *p/q*, which change the edge density of connecting edges (between graph *G* and graph *F*) from *p* ( = *p/q* × *q*) to *p*′ ( = *p*′*/q* × *q*).

Furthermore, we implemented GA with revised *p* model (GA + R model) to adjust a total edge density to be *p*. As represented in the grouped attachment explanation, the procedures only guarantee a density of edges connecting the existing graph with the added graph (edges between the graph *G* and the graph *F*) to be *p*. On the other hand, a density of edges of an added graph (the Graph *F*) is independent of *p*. As our model is designated to have a total edge density as *p* (like the ER model), we adjusted a density of connecting edges (edges between the graph *G* and the graph *F*) to be *p*′, which guarantees a total edge density to be *p* (Fig. 5). We deduced *p*′ by calculating an edge density of the added region (edges in the graph *F* and connecting region between the graph *G* and *F*) for each repeat (see Supplementary Note for *p*′ calculation).

### Topological property of GA model networks

To validate our models, we computed RGF-distances^25^ between a canonical Wnt signaling pathway in the NCI/Nature database (the pathway used in the introduction session) and its corresponding model networks of existing network models (ER, SW and SF) and our models (GA and GA + R) (see Supplementary Fig. S7). Creation processes of corresponding model networks of existing models are described in the Method session. RGF-distance compares the frequencies of the graphlets in two networks. To find the optimized *q* values for our models, we applied various *q* values ranging from 0.1 to 0.9 with an interval 0.1 (see Supplementary Note). Furthermore, we performed the same experiments to various types of undirected networks from Network Repository^36^; co-authorship network of scientists, airport network among cities, and real world road network (see Supplementary Note).

As described ahead, Fig. 1 shows directed 3-node motif frequency distributions of a canonical Wnt signaling pathway and its corresponding model networks of the existing network models (ER, SW and SF) and our models (GA and GA + R) with optimized *q* values (0.8 and 0.8, respectively). None of the existing network models reproduced a motif frequency distribution of the real network. On the other hand, GA models show similar shapes (high frequency at motif No. 3, 5, 6, 7, 10 and 11) of directed 3-node motif frequency distributions with the real network (purple lines in Fig. 1).

Supplementary Table S1 shows RGF-distances between canonical Wnt signaling pathway and its corresponding model networks of the existing network models (ER, SW and SF) and our models (GA and GA + R). Having low RGF-distances can be interpreted as they have more similar graphlet frequencies to the real-world network. GA models with optimized *q* values (0.8 and 0.8, respectively) show better results (lower values) in RGF-distances compared to the existing network models.

According to the results of motif frequency distributions and RGF-distances, our models showed better performances compared to the existing network models. The importance of the results is implied by the similar patterns (high frequency at motif No. 3, 5, 6, 7, 10 and 11) of 3-node motif frequency distribution of GA models with the real network, while the existing network models showed different aspects. GA models had better performances compared to the existing network models not only on the RGF-distances, but also on the aspects of 3-node motif frequency distribution. These results indicate that GA models reproduce motif properties of the real network better than other models.

Table 1 shows RGF-distances and co-neighborness values between various real-world networks (canonical Wnt signaling pathway, co-authorship of scientists, airport network among cities, and real-world road network) and their corresponding model networks of the existing network models (ER, SW, SF, and PSO/nPSO) and GA models. For PSO/nPSO models, the best performed results are shown among various input parameter settings (T = 0.1, 0.5, and 0.9; C = 0, 4, and 8; See method session for details). Except for the Inf_USAir network, GA models outperformed in RGF-distances. For the Inf_USAir network, GA models might generate better results for *q* value over 0.9, which was not included in the experiment.

**Table 1:** RGF-distance, co-neighborness and assortativity analysis results of various real-world networks.

	Canonical_wnt	Ca_netscience	Inf_USAir	Inf_euroroad
**Real-world network**				
Co-neighborness	0.19	0.30	0.26	0.01
Assortativity coefficient γ	−0.24	−0.08	−0.21	0.13
**Corresponding model (existing) networks**				
ER model				
RGF-distance	107.26 ± 4.51	112.89 ± 3.32	109.97 ± 3.74	75.26 ± 6.00
Co-neighborness	0.01 ± 0.00	0.00 ± 0.00	0.02 ± 0.00	0.00 ± 0.00
Assortativity coefficient γ	0.00 ± 0.03	−0.01 ± 0.03	0.00 ± 0.02	−0.01 ± 0.03
SW model				
RGF-distance	46.32 ± 2.79	72.90 ± 1.27	**21.01** ± **0.40**	102.11 ± 2.63
Co-neighborness	**0.24** ± **0.01**	**0.17** ± **0.01**	**0.32** ± **0.01**	0.00 ± 0.00
Assortativity coefficient γ	0.00 ± 0.04	0.00 ± 0.04	0.00 ± 0.02	0.01 ± 0.02
SF model				
RGF-distance	56.47 ± 4.07	118.56 ± 5.71	50.92 ± 1.55	131.14 ± 5.67
Co-neighborness	0.02 ± 0.00	0.02 ± 0.01	0.04 ± 0.00	0.00 ± 0.00
Assortativity coefficient γ	**−0.85** ± **0.05**	**−0.39** ± **0.06**	**−0.81** ± **0.03**	**−0.54** ± **0.11**
nPSO model				
Optimized T value and C value	0.1, 8	0.1, 8	0.1, 8	0.1, 8
RGF-distance	28.64 ± 1.28	68.92 ± 1.75	34.61 ± 1.13	98.73 ± 0.16
Co-neighborness	**0.13** ± **0.00**	0.08 ± 1.75	**0.19** ± **0.01**	0.00 ± 0.00
Assortativity coefficient γ	**−0.10** ± **0.02**	**−0.19** ± **0.03**	−0.09 ± 0.01	**−0.24** ± **0.03**
**Corresponding model (GA) networks**				
GA model				
Optimized q value	0.8	0.9	0.9	0.2
RGF-distance	22.97 ± 1.43	16.72 ± 2.85	33.30 ± 3.31	31.94 ± 10.14
Co-neighborness	**0.14** ± **0.01**	**0.19** ± **0.01**	**0.14** ± **0.01**	0.02 ± 0.00
Assortativity coefficient γ	0.00 ± 0.04	−0.05 ± 0.05	−0.07 ± 0.03	0.04 ± 0.03
GA + R model				
Optimized q value	0.8	0.9	0.9	0.2
RGF-distance	25.91 ± 2.28	**13.72** ± **2.66**	**32.33** ± **2.22**	**27.29** ± **17.86**
Co-neighborness	**0.14** ± **0.01**	**0.20** ± **0.01**	**0.15** ± **0.01**	0.01 ± 0.00
Assortativity coefficient γ	0.05 ± 0.06	0.02 ± 0.09	−0.02 ± 0.04	0.04 ± 0.03
GA + P model				
Optimized q value	0.9	0.9	0.9	0.1
RGF-distance	**20.87** ± **2.73**	52.99 ± 8.31	40.75 ± 2.29	87.74 ± 15.66
Co-neighborness	**0.17** ± **0.01**	**0.18** ± **0.01**	**0.15** ± **0.01**	0.01 ± 0.00
Assortativity coefficient γ	**−0.31** ± **0.03**	**−0.17** ± **0.03**	**−0.39** ± **0.02**	**−0.18** ± **0.02**
GA + RP model				
Optimized q value	0.6	0.9	0.9	0.1
RGF-distance	29.23 ± 4.88	43.73 ± 15.83	43.86 ± 2.80	67.83 ± 11.02
Co-neighborness	0.09 ± 0.01	**0.19** ± **0.01**	**0.16** ± **0.01**	0.01 ± 0.00
Assortativity coefficient γ	**−0.32** ± **0.04**	**−0.15** ± **0.02**	**−0.34** ± **0.03**	**−0.16** ± **0.02**

The table shows RGF-distance, co-neighborness, assortativity coefficient γ values of four real-world networks and their corresponding model networks. For models, which require optimization, also stated the optimized parameter(s). Average values and standard deviations are stated together as the experiments are performed 10 times (100 times for canonical_wnt) per every condition, and averaged the results. Low RGF-distance represents high motif similarity to real-world networks. Co-neighborness is our proposed measure, which is highly related to common neighbors and community/cluster structures. Low assortativity coefficient γ values are related to scale-free property. Best performed RGF-distances, relatively high co-neighborness values, and relatively low assortativity coefficient γ values are stated in bold.

For three real-world networks (Canonical_wnt, Ca_netscience, and Inf_USAir) which have high co-neighborness values (0.19, 0.30, and 0.26, respectively), GA models showed relatively high co-neighborness values compared to ER and SF model. SW and PSO/nPSO models also showed relatively high co-neighborness values, because SW and PSO/nPSO model generates networks with high clustering coefficients, and clusters increase co-neighborness values of the network. Inf_euroroad network has a low co-neighborness value (0.01) and all model networks showed low co-neighborness values. As optimized *q* values for GA models of Inf_euroroad are also low, there seems to be a correlation between co-neighborness and optimized *q* values, which might be a good following research topic. In general, corresponding model networks of similar co-neighborness values with real-world networks showed low RGF-distances, which implies that co-neighborness can be a good topological measure of network motifs.

From these results, we can insist that GA models generate networks which have high topological similarities with real-world networks in the manner of RGF-distances. As well, co-neighborness showed its potential to be a representing topological measure of network motifs.

### Preferential GA models (GA + P and GA + RP models)

**Figure 6:**
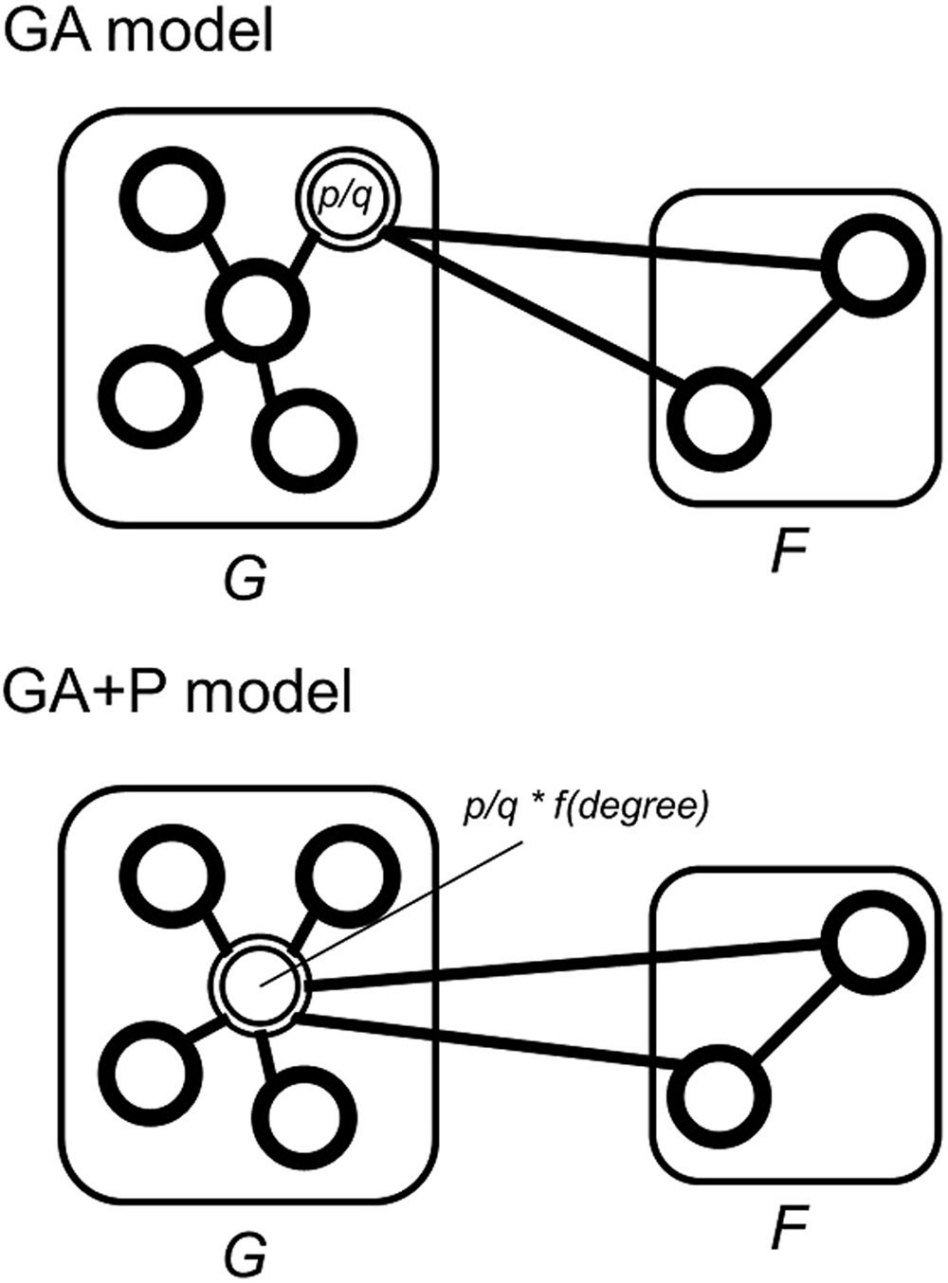
Edge generation procedures of GA and the preferential attachment adapted model (GA + P). The figure shows an example of edge generation procedures (Step 3 of Fig. 4) of both models. High degree nodes have higher probability to be selected in attachment procedure of GA + P model. GA + P model shows a scale-free property while maintaining a motif property of our model.

As the scale-free model (SF model) has been widely analyzed and represented as a proper network model in various types of networks^3,37,38^, we applied the preferential attachment procedures to our models. The preferential GA model (GA + P model) is a combined model which preferentially attaches nodes when connecting the added graph (Graph *F*) with the existing graph (Graph *G*) (Fig. 6). During the attachment procedure (step 3 in Fig. 4), we select $|{V}_{G}|\cdot p/q$ nodes depending on the distribution $deg{({V}_{G})}^{{\alpha }}+a$, where *deg*(*V*_*G*_) indicates a degree (in-degree for a directed graph) distribution of nodes in the Graph *G*, *α* indicates power of preferential attachment, and a indicates initial attractiveness of the nodes^3^. This procedure guarantees higher connection probabilities on nodes of higher degrees, while preserving a motif property of our model. Furthermore, we also implemented GA + RP model, which adapted preferential attachment to GA + R model.

**Figure 7:**
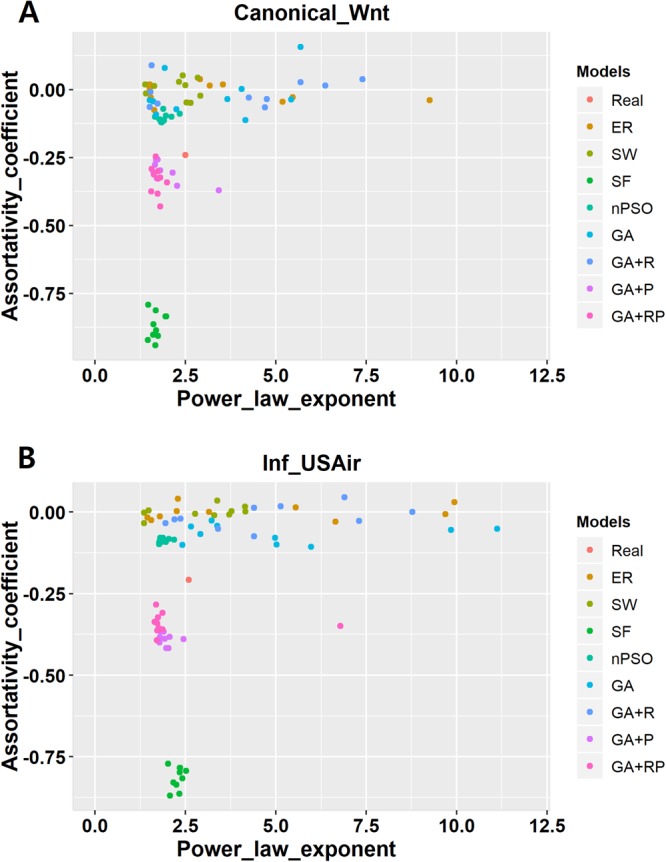
Assortativity coefficients and power law exponents of two real-world networks and their corresponding model networks. Two real-world networks, (**A**) Canonical_Wnt and (**B**) Inf_USAir, are selected as both of them have power law exponents between two and three, which represents scale-free property. Assortativity coefficients and power law exponents are measured from those networks and their corresponding model networks. For PSO/nPSO and GA models, we used the optimized input parameters stated in the Table 1. Most networks with low negative assortativity coefficients have power law exponents around two and three, which represents scale-free property. There exist couple of outliers in GA + P and GA + RP model networks.

To show preferential attachment procedures are well-implemented in preferential models (GA + P and GA + RP models), we computed RGF-distances^25^, power law exponents^3^, and assortativity coefficients^39^ of the networks. Scale-free networks have degree distributions following power law with exponents in the range between two and three^3^. As some of network generation models get power law exponents as one of their input parameters, we also measured assortativity coefficients as indirect measurements of scale-free property^40^. Power law exponents and assortativity coefficients are measured from two real-world networks (Canonical_Wnt and Inf_USAir) and their corresponding model networks (Fig. 7). Networks generated by SF, PSO/nPSO, GA + P, and GA + RP have low assortativity coefficients and power law exponents around two to three, which represents those networks are scale-free and confirms that low negative assortativity coefficients are related to scale-free properties. In Table 1, networks generated by SF and PSO/nPSO models show low negative values of assortativity coefficient *r*, while networks generated by ER and SW models show *r* values close to 0. This result also supports that scale-free properties are represented with low negative values of *r*. As networks generated by preferential models (GA + P and GA + RP models) have power law exponents around two to three and show relatively low negative values of *r* compared to networks generated by non-preferential models (GA and GA + R models), we can claim that networks generated by preferential models have scale-free properties.

Furthermore, it is notable that RGF–distances are quite similar between the non-preferential models (GA and GA + R models) and the preferential models (GA + P and GA + RP models) in canonical Wnt signaling pathway and airport network among cities, while preferential models had poor RGF-distances compared to non-preferential models in other networks (co-authorship network of scientists and real world road network). As real-world networks of canonical Wnt signaling pathway and airport network among cities showed relatively low negative values (−0.24 and −0.21, respectively) of *r*, we can assume that those two networks have scale-free properties. Then, we can conclude that the preferential models showed good performances of motif properties in real-world networks with scale-free properties.

According to these results, we can claim that preferential models (GA + P and GA + RP) gained scale-free properties while preserving real-world motif properties. Also, it can be implied that preferential attachment procedures are well-implemented in preferential models (GA + P and GA + RP).

## Discussion

In summary, we suggested a new network measure, co-neighborness, and a new network model, grouped attachment, to represent real-world network topologies. We showed that some of real-world networks have high co-neighborness, and reproducing the co-neighborness can generate real-world topologies. As the preferential attachment is suggested to reproduce scale-free properties of real-world networks^3^, we suggested the grouped attachment to reproduce co-neighborness of real-world networks. By applying the grouped attachment to random network generation, we have developed a new network model which has higher similarities of motif properties with real-world networks. While existing network models could not reproduce motif frequency distribution of real-world networks, our proposed model showed higher similarities of motif frequencies with real-world networks quantitatively. Furthermore, we applied preferential attachment procedures to our model, to gain scale-free properties while preserving real-world motif properties.

Nevertheless, existing network models have their capability to reproduce some of real-world properties. While our models outperformed on reproducing network motif properties, existing models have their own specialties on different real-world properties; SF model for scale-free properties; SW model for small world properties; PSO/nPSO models for various properties stated in the introduction session. It would be responsible for users to choose appropriate models for a given task.

As co-neighborness adopted the concept of Jaccard’s index, graphs with high co-neighborness would have pairs of adjacent nodes which are located closely in a hidden geometric space^10^. Furthermore, if you apply co-neighborness (Jaccard’s index) concept to the community/cluster structure, you can interpret it as “A pair of nodes in the community/cluster structure would likely to have similar common neighbors, so that they have high interconnections in the community/cluster and few connections to nodes out of the community/cluster”. In this interpretation, we have focused on ‘few connections to nodes out of the community/cluster’. We thought that not only the fewness of connections is important, but also those few connections should be (likely to be) connected to the same nodes (out of the community/cluster), not randomly. This idea is well implemented in our grouped attachment model. We assumed the highly interconnected graph *F* represents a community/cluster. When they attach to existing graph *G*, they make connections to specifically selected nodes (not to random nodes). These implementations led our model to have reproducibility of neighborhood similarities.

In-depth analysis of co-neighborness and optimization of *q* values can be a good candidate following research topics. Our findings show that real-world complex networks are populated by topological motifs and the proposed model reproduces real-world topological properties. These findings can be applied to various network topology studies such as community detection^41^ and link prediction^42,43^. Some of existing methods of both tasks, community detection and link prediction, are dependent on network models which reproduce real-world topologies. They use network models for network structure estimation, and infer results based on them. As our proposed GA models uniquely reproduces real-world motif properties, implementing our models might be a key solution to the tasks.

## Methods

### Common neighbor index and Jaccard index

Let *u* and *v* are network nodes, and *Γ(u)* and *|Γ(u)|* refer to the set of neighbors of *u* and the cardinality of the set, respectively. Common neighbor index^26^ is defined as

2 $$CN(u,v)=|\Gamma (u){\cap }^{}\Gamma (v)|,$$

and Jaccard index^33,34^ is defined as

3 $$JC(u,v)=\frac{|\Gamma (u){\cap }^{}\Gamma (v)|}{|\Gamma (u){\cup }^{}\Gamma (v)|}=\frac{CN(u,v)}{|\Gamma (u){\cup }^{}\Gamma (v)|}.$$

### Random network generation of existing models

All random networks of existing models (ER, SW, and SF) are generated through ‘igraph’ R package (package version 1.0.1)^44^. To generate input parameters of network models, we measure *|N|* (number of nodes), *|E|* (number of edges), *p* (edge density), *α* (exponent of the fitted power-law distribution of degree), and *a* (minimum value from which the power-law distribution of degree was fitted) from a given real-world network.

For ER network generation, we adapted Erdös–Rényi model by utilizing erdos.renyi.game() function in ‘igraph’ package. We set the number of nodes to be *|N|*, and the edge probability to be *p*.

For SW network generation, we adapted Watts-Strogatz model by utilizing watts.strogatz.game() function in ‘igraph’ package. We set the dimension of the starting lattice to be 1, the size of the lattice along each dimension to be *|N|*, the neighborhood within which the vertices of the lattice will be connected to be *|E|/|N|*, and the rewiring probability to be 0.05.

For SF network generation, we adapted Barabasi-Albert (preferential attachment) model by utilizing barabasi.game() function in ‘igraph’ package. We set the number of vertices to be *|N|*, the power of the preferential attachment to be *α*, the number of edges to add in each time step to be *|E|/|N|*, and the attractiveness of the vertices with no adjacent edges to be *a*.

For PSO/nPSO network generation, we adapted nPSO model in the corresponding manuscript^27^. We set the number of nodes to be *|N|*, the half of average degree to be *|E|/|N|*, the exponent of the power-law node degree distribution to be *α*. The random networks of PSO/nPSO model were generated with three different temperature values (T = 0.1, 0.5, and 0.9), and three different numbers of communities (C = 0, 4, and 8); C = 0 corresponds to the PSO model, while C = 4, 8 corresponds to the nPSO model.

Using each model, we generated 100 random networks (100 repeats) for directed networks and 10 random networks (10 repeats) for undirected networks. All experiments are performed to the networks and the results are averaged.

### Counting motif frequencies

Counting network motif frequencies have been processed differently depending on directedness of the given network. For directed networks, we utilized graph.motifs() function in ‘igraph’ R package^44^. For undirected networks, we utilized countMotif() function in ‘NeMo’ R package (package version 1.0.1)^45^.

### RGF-distance calculation

RGF-distance compares the frequencies of the appearance of all 3–5-node graphlets in two networks^13^. Between two graphs *G* and *H*, RGF-distance is defined as

4 $$D(G,\,H)={\sum }_{i=1}^{29}|{F}_{i}(G)-{F}_{i}(H)|,$$

where

5 $${F}_{i}(G)=-log(\frac{{N}_{i}(G)}{T(G)}).$$

*N*_*i*_(*G*) is the number of graphlets (motif frequency count) of type *i* (*i* ∈ {1, …, 29} for graphlet size from 3 to 5) in a network *G*, and

6 $$T(G)={\sum }_{i=1}^{29}{N}_{i}(G)$$

is the total number of graphlets of *G*. Graphlet types can be referred to motif numbers in network motifs. In our experiments, we computed RGF-distances between real-world networks and their corresponding model networks.

### Power law exponent measurement

Power law exponent *k* is a measure of scale-free property. Degree distribution of a scale-free network follows power law with exponent 2 < *k* < 3^3^. Power law exponent *k* of network is measured through power.law.fit() function in ‘igraph’ R package (package version 1.0.1)^44^.

### Assortativity coefficient measurement

Assortativity coefficient *r* is a measure of the likelihood for nodes to connect to other nodes with similar degrees^39^, and is related to a scale-free metric^40^. Assortativity coefficient ranges between −1 and 1. When *r* = 1, the network is completely assortative. When *r* = 0, the network is non-assortative. When *r* = −1, the network is completely disassortative. Assortativity coefficient *r* of network is measured through assortativity.degree() function in ‘igraph’ R package (package version 1.0.1)^44^.

### Code availability

The R script implementing the GA models and the co-neighborness is available at https://github.com/bisl-kaist/GA_model.

## Electronic supplementary material

Supplementary Information

Supplementary Table S4

## Data Availability

The Canonical Wnt signaling pathway data analyzed during the current study are available in the NCI/Nature database by ‘import network from web services’ in Cytoscape^46^. The Co-authorship of scientists^47^, the airport network among cities^48^, and the real-world road network data^49^ analyzed during the current study are available in the Network Repository, http://networkrepository.com/^36^. Detailed descriptions of four networks are stated in Supplementary Note.
