# Supplementary material for: Topological motifs populate complex networks through grouped attachment
Source: Sci Rep. 2018 Aug 23;8:12670. doi: 10.1038/s41598-018-30845-4 (PMC6107624; doi:10.1038/s41598-018-30845-4)
Supplement: Supplementary file 1 — Supplementary Information [file 41598_2018_30845_MOESM1_ESM.pdf]

Supplementary Information

Title: Topological motifs populate complex networks through grouped attachment

Authors and affiliations
Jaejoon Choi[1,2], Doheon Lee[1,3*]

[1]Bio-Synergy Research Center, 291 Daehak-ro, Yuseong-gu, Daejeon, Republic of Korea
[2]Department of Genetics, Harvard Medical School, 77 Avenue Louis Pasteur, Boston, Massachusetts, United States of America
[3]Department of Bio and Brain Engineering, Korea Advanced Institute of Science and Technology (KAIST), 291 Daehak-ro, Yuseong-gu, Daejeon, Republic of Korea

Supplementary Notes

Groupness probability q value analysis

Our proposed models have three input parameters; n, p, and q (GA+P and GA+RP models require more parameters for preferential attachment procedures). Groupness probability q is proposed as a new parameter, while other two input parameters, n and p, have been widely used in conventional network models. Groupness probability q represents how the network is co-neighbored, and ranges from p to 1. q has to be larger than p, because probability p/q cannot exceed 1 during network generation.

Optimized q values differ depending on network properties of corresponded networks (Supplementary Fig. S5). If a corresponded network is an ER model-like network, the optimized q value is supposed to be very low (close to 0). If q value is close to 0, network generation procedure of our model generates similar results with ER model. In Table 1, real world road network (Inf_euroroad) are assumed to be ER-model-like network, because it was the only network which has the lowest RGF-distance score with ER model among three existing models (ER, SW, and SF models). Surprisingly, optimized q values of the network are close to 0. If a corresponded network has small world property, the optimized q value appears to be very high (close to 1). In Table 1, the co-authorship network of scientists (Ca_netscience) and the airport network among cities (Inf_USAir), which both are well-known small world networks[1,2], have their optimized q values close to 1. Predicting an optimized q value for a given network and finding correlations between the q value and other network metrics including co-neighborness are supposed to be challenging and worthy research issues for a development of our proposed model.

Edge extension model

In the grouped attachment (GA) model, subgraphs are generated with the edge expansion model. The edge expansion model is proposed by authors in this paper, which has following characteristics: 1) The model generates connected graph, 2) Generated graph size is proportional to an input parameter q.

Generation processes of the edge expansion model are illustrated at Supplementary Fig. S6. The model gets one input parameter q, which has a range from 0 to 1. The generation starts with a single node graph. Among all possible edges (edges between nodes which are unconnected currently and edges between one of existing node and newly created node), one edge is selected in random (equal probability).

The selected edge (and the new node, if created) is created with probability q. If the edge is created, repeat the creation process, and if the edge is not created, terminate the creation. A connected graph, which has its size proportional to an input parameter q is generated with the model.

The edge expansion model has been proposed as a subnetwork generation model (Supplementary Fig. S6), because a network generation model with a single input value (q value) is required for a simplification of the model. The subnetwork is required to have large amount of nodes and edges proportional to the input value. When any network generation model which satisfies the requirement is adopted as a subnetwork generation model, it is expected to generate similar results.

Revised calculation of *p* to *p'* in GA with revised *p* model (GA+R model)

For an undirected graph,

$$\begin{aligned}
&\text{Edge density} \\
&= \frac{\text{existing edges}}{\text{possible edges}} \\
&= \frac{\text{e. edges(graph F)} + \text{e. edges(connecting)}}{\text{p. edges(graph F)} + \text{p. edges(connecting)}} \\
&= \frac{|E_F| + p'|V_G||V_F|}{\frac{|V_F|(|V_F|-1)}{2} + |V_G||V_F|} \\
&= p \\
&\therefore \text{p}' = \frac{p\left(\frac{|V_F|(|V_F|-1)}{2} + |V_G||V_F|\right) - |E_F|}{|V_G||V_F|}.
\end{aligned}$$

For a directed graph,

$$\begin{aligned}
&\text{Edge density} \\
&= \frac{\text{existing edges}}{\text{possible edges}} \\
&= \frac{\text{e. edges(graph F)} + \text{e. edges(connecting)}}{\text{p. edges(graph F)} + \text{p. edges(connecting)}} \\
&= \frac{|E_F| + p'2|V_G||V_F|}{|V_F|(|V_F|-1) + 2|V_G||V_F|} \\
&= p \\
&\therefore p' = \frac{p(|V_F|(|V_F|-1) + 2|V_G||V_F|) - |E_F|}{2|V_G||V_F|}
\end{aligned}$$

where $|V_G|$ indicates a number of nodes in the existing graph (graph *G*), $|V_F|$ indicates a number of nodes in the added graph (graph *F*), and $|E_F|$ indicates a number of edges in the added graph (graph *F*).

Real-world networks used in the experiments

Canonical Wnt signaling pathway (Canonical_wnt) is downloaded from NCI / Nature database by utilizing 'import network from web services' in Cytoscape[3]. It is a directed graph with 248 nodes and 791 edges (Supplementary Fig. S1).

Co-authorship of scientists (Ca_Netscience)[4] is downloaded from Network repository (http://networkrepository.com/)[5]. It is an undirected graph with 379 nodes and 914 edges (Supplementary Fig. S2).

Airport network among cities (Inf_USAir)[6] is downloaded from Network repository (http://networkrepository.com/)[5]. It is an undirected graph with 332 nodes and 2,126 edges (Supplementary Fig. S3).

Real world road network (Inf_Euroroad)[7] is downloaded from Network repository (http://networkrepository.com/)[5]. It is an undirected graph with 1,174 nodes and 1,417 edges (Supplementary Fig. S4).

Animal behavior network (Cattle)[8] is downloaded from Koblenz Network Collection (http://konect.uni-koblenz.de). It is a directed graph with 28 nodes and 217 edges.

Animal social network (Dolphin)[9] is downloaded from Koblenz Network Collection (http://konect.uni-koblenz.de). It is a directed graph with 62 nodes and 159 edges.

Friendship network (Highschool)[10] is downloaded from Koblenz Network Collection (http://konect.uni-koblenz.de). It is a directed graph with 70 nodes and 366 edges.

Karate club network (Karate)[11] is downloaded from Koblenz Network Collection (http://konect.uni-koblenz.de). It is an undirected graph with 34 nodes and 78 edges.

Gift-giving network (Taro_Exchange)[12,13] is downloaded from Koblenz Network Collection (http://konect.uni-koblenz.de). It is a directed graph with 22 nodes and 78 edges.

Animal interaction network (Zebra)[14] is downloaded from Koblenz Network Collection (http://konect.uni-koblenz.de). It is an undirected graph with 27 nodes and 111 edges.

Refer Supplementary Table S2 for basic network statistics of the first four networks. RGF-distance analysis results for the last six networks are in Supplementary Table S4 (independent excel file).

Comparison of co-neighborness with other network measures

The new network measure, co-neighborness, has been compared with other network measures. We selected 15 different popular network measures for a comparison. The list of all measures is as below:

- Conei : co-neighborness
- Ncount : number of nodes; measured through vcount() function in ‘igraph’ R package (package version 1.0.1)[15]
- Ecount : number of edges; measured through ecount() function in ‘igraph’ R package (package version 1.0.1)[15]
- Density : edge density of a graph; measured through graph.density() function in ‘igraph’ R package (package version 1.0.1)[15]
- Triangle_cnt : total number of triangles; measured through count_triangles() function in ‘igraph’ R package (package version 1.0.1)[15]
- Triangle_avr : average number of triangles; measured through count_triangles() function in ‘igraph’ R package (package version 1.0.1)[15]
- Triangle_max : maximum number of triangles; measured through count_triangles() function in ‘igraph’ R package (package version 1.0.1)[15]
- Cluster_glo : global clustering coefficient; measured through transitivity() function in ‘igraph’ R package (package version 1.0.1)[15]

- Cluster_avr : average clustering coefficient; measured through transitivity() function in 'igraph' R package (package version 1.0.1)[15]
- Assortativity : assortativity coefficient; measured through assortativity.degree() function in 'igraph' R package (package version 1.0.1)[15]
- Closeness : closeness centrality; measured through closeness() function in 'igraph' R package (package version 1.0.1)[15]
- BC_edge : betweeness centrality; measured through betweenness() function in 'igraph' R package (package version 1.0.1)[15]
- BC_node : betweeness centrality; measured through edge_betweenness() function in 'igraph' R package (package version 1.0.1)[15]
- Modularity : modularity; measured through modularity() function in 'igraph' R package (package version 1.0.1)[15]
- LCP_corr : local-community-paradigm (LCP) correlation; measured based on the source code in from the literature[16]
- Richness : richness p-value; measured based on the source code in from the literature[17]

Network measures are measured for 10 different real-world networks stated in 'Real-world networks used in the experiments'. Supplementary Table S3 provides correlations among network measures.

There are several existing network measures which are related to co-neighborness such as graph density or average/global clustering coefficient. These measures can have high correlation with co-neighborness, but have different values as co-neighborness has a different definition.

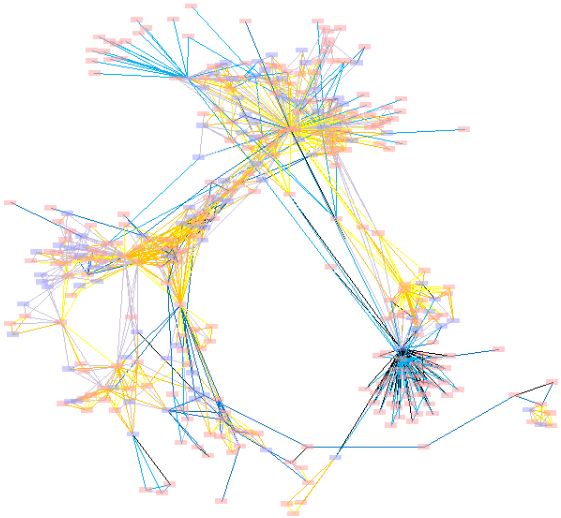

Supplementary Figure S1 | Canonical Wnt signaling pathway in the NCI / Nature database A directed graph with 248 nodes and 791 edges is displayed by Cytoscape. Colors of nodes and edges represent node types and edge types, respectively.

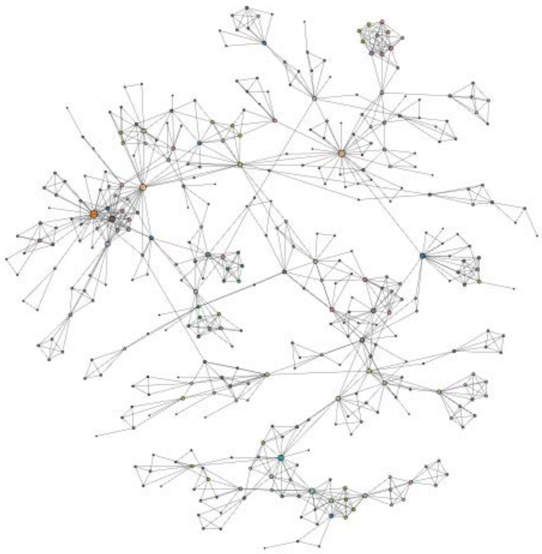

Supplementary Figure S2 | Co-authorship of scientists An undirected graph with 379 nodes and 914 edges.

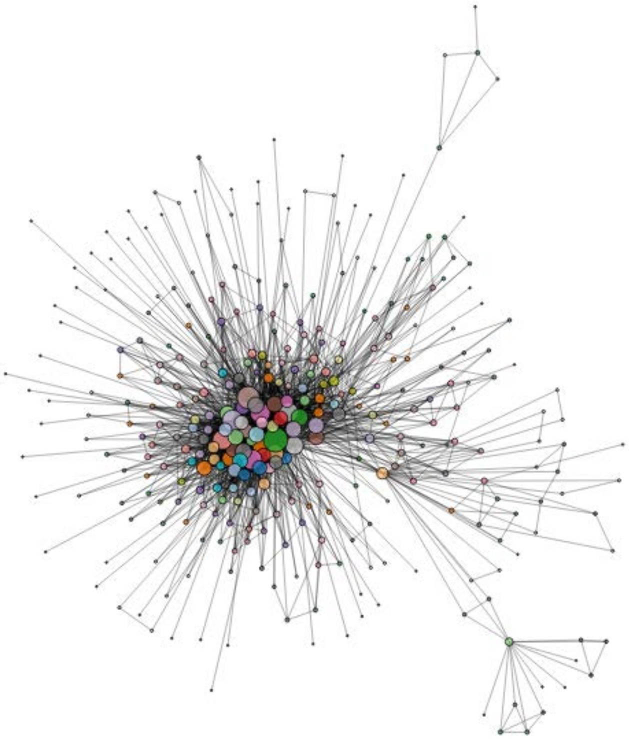

Supplementary Figure S3 | Airport network among cities An undirected graph with 332 nodes and 2,126 edges.

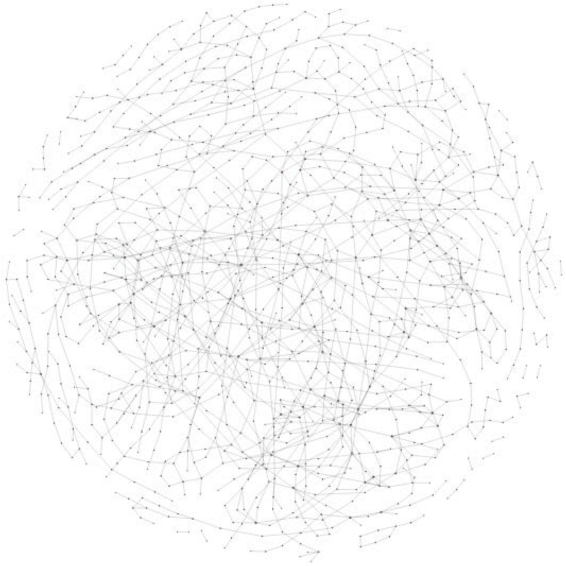

Supplementary Figure S4 | Real world road network An undirected graph with 1,174 nodes and 1,417 edges.

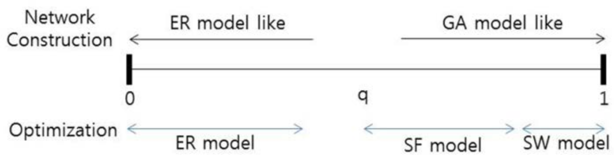

Supplementary Figure S5 | Groupness probability q value range If a corresponded network is an ER model-like network, the optimized q value is supposed to be very low (close to 0). If q value is close to 0, network generation procedure of our model generates similar results with ER model. If a corresponded network has small world property, the optimized q value appears to be very high (close to 1).

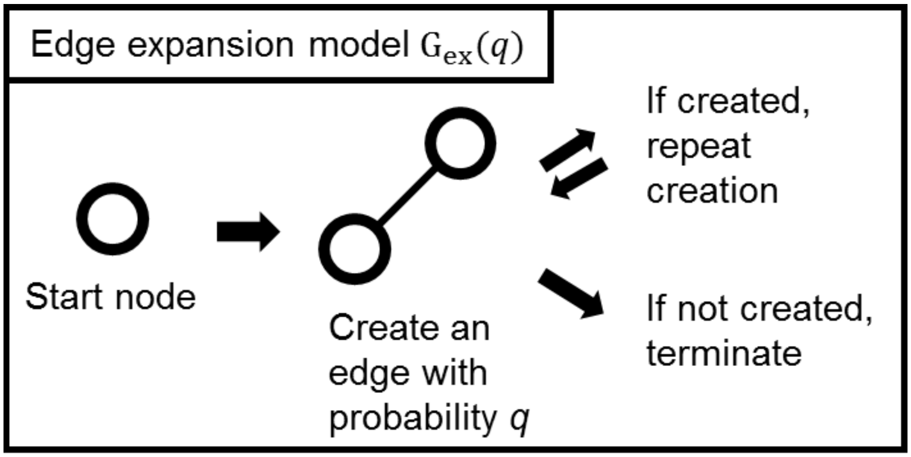

Supplementary Figure S6 | Edge expansion model A subgraph generation model used in GA model generation. Detailed description is stated in Supplementary Notes.

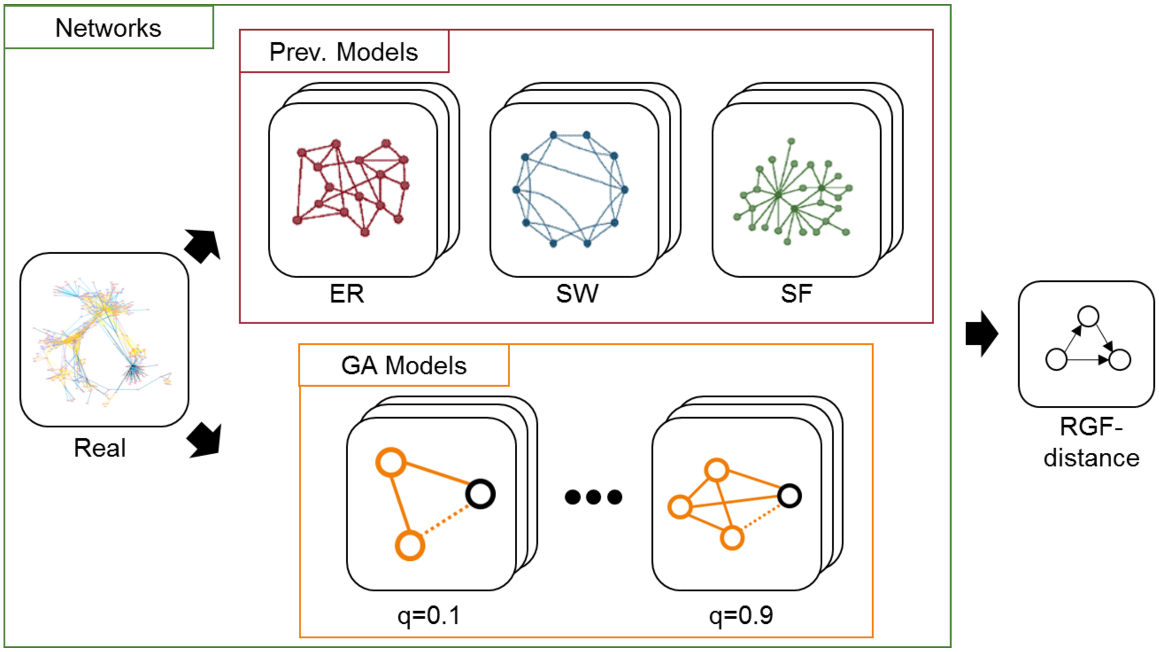

Supplementary Figure S7 | Experiment design for validation We calculated RGF-distance between canonical Wnt signaling pathway in the NCI / Nature database and corresponding model networks of existing network models (ER, SW and SF) and our models (GA, GA+R). To find the optimized q values for our models, we applied various q values ranging from 0.1 to 0.9 (interval=0.1).

Supplementary Table S1 | RGF-distance analysis result of canonical Wnt signaling pathway

		Existing models			GA models	
		ER	SW	SF	GA	GA+R
q	0.1	**107.1760**	**46.0005**	**57.1890**	104.7612	104.9284
	0.2				92.0677	95.5564
	0.3				76.9334	79.5752
	0.4				60.7662	63.2644
	0.5				46.3683	50.2427
	0.6				35.9180	38.7905
	0.7				26.1205	28.9222
	0.8				**22.9704**	**25.9060**
	0.9				25.6166	29.8084

RGF-distances are calculated between canonical Wnt signaling pathway (undirected network) and its corresponding model networks of existing network models (ER, SW, and SF) and our models (GA and GA+R). Various q values ranging from 0.1 to 0.9 (interval=0.1) were applied to our models.

Supplementary Table S2 | Network statistics of real-world networks

	Canonical_wnt	Ca_netscience	Inf_USAir	Inf_euroroad
**Count**				
Nodes	248	379	332	1174
Edges	791	914	2126	1417
**Network Measures**				
Density	0.0258	0.0128	0.0387	0.0021
Assortativity coefficient γ	-0.2413	-0.0817	-0.2079	0.1267
**Degree**				
Maximum	117	34	139	10
Minimum	1	1	1	1
Average	6.3790	4.8232	12.8072	2.4140

Basic network statistics are measured for real-world networks which were used in the experiments.

Supplementary Table S3 | Comparison of co-neighborness with other network measures

	Mean	St.dev	Corr_conei
**Network measures**			
Conei	0.244	0.171	1
Cluster_glo	0.378	0.231	0.975
Cluster_avr	0.555	0.269	0.764
Density	0.144	0.169	0.668
LCP_corr	0.728	0.390	0.636
BC_node	11.715	11.967	-0.620
Assortativity	-0.063	0.332	0.604
Modularity	0.473	0.244	-0.569
Ncount	237.6	356.059	-0.480
Richness	0.300	0.354	-0.466
Triangle_avr	26.027	36.877	0.466
BC_edge	34.861	71.688	-0.238
Ecount	611.4	700.242	-0.233
Closeness	0.008	0.009	0.193
Triangle_max	209.1	432.161	0.087
Triangle_cnt	4682.7	11250.83	0.066

Mean and standard deviation value of each network measures are measured from 10 real-world networks. Refer Supplementary Note for description of network measures. Correlation coefficients with co-neighborness are calculated and stated in the 'Corr_conei' column. Rows/columns are sorted by absolute value of correlation coefficient with co-neighborness.

Supplementary Table S4 | RGF-distance, co-neighborness and assortativity analysis results of additional real-world networks

RGF-distance, co-neighborness and assortativity analysis were performed to six additional real-world networks. Average values and standard deviations are stated together as the experiments are performed 10 times per every condition, and averaged the results. Best performed RGF-distances are stated in bold.

References

1 Amaral, L. A. N., Scala, A., Barthelemy, M. & Stanley, H. E. Classes of small-world networks. *Proceedings of the national academy of sciences* **97**, 11149-11152 (2000).

2 Liu, X., Bollen, J., Nelson, M. L. & Van de Sompel, H. Co-authorship networks in the digital library research community. *Information processing & management* **41**, 1462-1480 (2005).

3 Smoot, M. E., Ono, K., Ruscheinski, J., Wang, P.-L. & Ideker, T. Cytoscape 2.8: new features for data integration and network visualization. *Bioinformatics* **27**, 431-432 (2011).

4 Newman, M. E. Finding community structure in networks using the eigenvectors of matrices. *Physical review E* **74**, 036104 (2006).

5 Rossi, R. A. & Ahmed, N. K. An Interactive Data Repository with Visual Analytics. *ACM SIGKDD Explorations Newsletter* **17**, 37-41 (2016).

6 Colizza, V., Pastor-Satorras, R. & Vespignani, A. Reaction–diffusion processes and metapopulation models in heterogeneous networks. *Nature Physics* **3**, 276 (2007).

7 Bader, D. A., Meyerhenke, H., Sanders, P. & Wagner, D. Graph partitioning and graph clustering, in *10th DIMACS Implementation Challenge Workshop.*

8 Schein, M. W. & Fohrman, M. H. Social dominance relationships in a herd of dairy cattle. *The British Journal of Animal Behaviour* **3**, 45-55 (1955).

9 Lusseau, D. *et al.* The bottlenose dolphin community of Doubtful Sound features a large proportion of long-lasting associations. *Behavioral Ecology and Sociobiology* **54**, 396-405 (2003).

10 Coleman, J. S. Introduction to mathematical sociology. *Introduction to mathematical sociology.* (1964).

11 Zachary, W. W. An information flow model for conflict and fission in small groups. *Journal of anthropological research* **33**, 452-473 (1977).

12 Hage, P. & Harary, F. *Structural models in anthropology*. (Cambridge University Press, 1983).

13 Schwimmer, E. *Exchange in the social structure of the Orokaiva: traditional and emergent ideologies in the Northern District of Papua*. (Hurst & Co., 1973).

14 Sundaresan, S. R., Fischhoff, I. R., Dushoff, J. & Rubenstein, D. I. Network metrics reveal differences in social organization between two fission–fusion species, Grevy's zebra and onager. *Oecologia* **151**, 140-149 (2007).

15 Csardi, G. & Nepusz, T. The igraph software package for complex network research. *InterJournal, Complex Systems* **1695**, 1-9 (2006).

16 Cannistraci, C. V., Alanis-Lobato, G. & Ravasi, T. From link-prediction in brain connectomes and protein interactomes to the local-community-paradigm in complex networks. *Scientific reports* **3**, 1613 (2013).

17 Muscoloni, A. & Cannistraci, C. V. Rich-clubness test: how to determine whether a complex network has or doesn't have a rich-club? *arXiv preprint arXiv:1704.03526* (2017).
